# Antibacterial Activity and Cytotoxicity Screening of Acyldepsipeptide-1 Analogues Conjugated to Silver/Indium/Sulphide Quantum Dots

**DOI:** 10.3390/antibiotics13020183

**Published:** 2024-02-13

**Authors:** Sinazo Z. Z. Cobongela, Maya M. Makatini, Bambesiwe May, Zikhona Njengele-Tetyana, Mokae F. Bambo, Nicole R. S. Sibuyi

**Affiliations:** 1 Health Platform, Advanced Materials Division, Mintek, Randburg 2194, South Africa; znjengele-tetyana@wrhi.ac.za; 2 Molecular Sciences Institute, School of Chemistry, University of the Witwatersrand, Johannesburg 2050, South Africa; maya.makatini@wits.ac.za; 3 Department of Science and Innovation (DSI)/Mintek Nanotechnology Innovation Centre (NIC), Advanced Materials Division, Mintek, Randburg 2194, South Africa; bambesiwem@mintek.co.za (B.M.); mokaeb@mintek.co.za (M.F.B.); 4 Institute for Nanotechnology and Water Sustainability (iNanoWS), College of Science, Engineering and Technology, University of South Africa, Florida Campus, Roodepoort 1705, South Africa; 5 Wits RHI, University of the Witwatersrand, Johannesburg 2050, South Africa; 6 Department of Science and Innovation (DSI)/Mintek Nanotechnology Innovation Centre (NIC), Biolabels Research Node, Department of Biotechnology, University of the Western Cape, Bellville 7535, South Africa

**Keywords:** acyldepsipeptides, antibacterial peptides, anionic peptides, palmitic acid, adamantane, nanomaterials, nano-carriers, AgInS_2_ quantum dots

## Abstract

The continuous rise in bacterial infections and antibiotic resistance is the driving force behind the search for new antibacterial agents with novel modes of action. Antimicrobial peptides (AMPs) have recently gained attention as promising antibiotic agents with the potential to treat drug-resistant infections. Several AMPs have shown a lower propensity towards developing resistance compared to conventional antibiotics. However, these peptides, especially acyldepsipeptides (ADEPs) present with unfavorable pharmacokinetic properties, such as high toxicity and low bioavailability. Different ways to improve these peptides to be drug-like molecules have been explored, and these include using biocompatible nano-carriers. ADEP1 analogues (SC005-8) conjugated to gelatin-capped Silver/Indium/Sulfide (AgInS_2_) quantum dots (QDs) improved the antibacterial activity against Gram-negative (*Escherichia coli* and *Pseudomonas aeruginosa*), and Gram-positive (*Bacillus subtilis*, *Staphylococcus aureus* and Methicillin-resistant *Staphylococcus aureus*) bacteria. The ADEP1 analogues exhibited minimum inhibition concentrations (MIC) between 63 and 500 µM, and minimum bactericidal concentrations (MBC) values between 125 and 750 µM. The AgInS_2_-ADEP1 analogue conjugates showed enhanced antibacterial activity as evident from the MIC and MBC values, i.e., 1.6–25 µM and 6.3–100 µM, respectively. The AgInS_2_-ADEP1 analogue conjugates were non-toxic against HEK-293 cells at concentrations that showed antibacterial activity. The findings reported herein could be helpful in the development of antibacterial treatment strategies.

## 1. Introduction

In recent years, nanotechnology has attracted significant interest, especially in the pharmaceutical industry. Nanomaterials have been explored as promising tools for biomedical applications such as biosensors, drug delivery, and imaging, amongst others [1]. Nanomaterials are very small, usually ranging from 1 to 100 nm, and have a larger surface area. Importantly, nanomaterials can be easily functionalized by conjugating compounds of interest [2]. Properties of nanomaterials such as size, chemical composition, shape, and surface structure can significantly influence how they interact with other molecules. For instance, the surface functionalization of core nanomaterials with various ligands can lead to their use in different biological applications. Due to their small size, nanomaterials can passively diffuse through cell membrane pores and ion channels via endocytosis. In addition, active targeting can be achieved by attaching ligands to the nanomaterials to facilitate internalization by specific cells [3]. Nanomaterials are therefore good drug delivery systems that can improve existing drugs to achieve desirable therapeutic efficacy. The conjugation of pharmaceutically active compounds with poor pharmacokinetic properties to nanomaterials has been shown to increase drug solubility, bioavailability, and decrease enzyme susceptibility [4,5,6]. Nanomaterials can reduce toxicity and adverse side effects of conventional drug molecules by increasing drug selectivity and improving permeability across membranes, including the blood–brain barrier [7].

Nanomaterial have also been shown to play a pivotal role in enhancing the antimicrobial activity of antimicrobial peptides (AMPs) [8]. AMPs are promising antibiotic agents with the potential to treat drug-resistant infections. Several AMPs have shown a lower propensity towards developing resistance compared to conventional antibiotics [9]. However, these peptides, especially acyldepsipeptides (ADEPs) present with unfavourable pharmacokinetic properties such as high toxicity and low bioavailability [10]. ADEPs are a class of AMPs that target the bacterial ClpP protease and have great potential as antibiotics. The ADEPs bind and dysregulate the bacterial caseinolytic protease (ClpP), which is responsible for the overall bacterial cell protein homeostasis. Different ways to improve ADEPs to be drug-like molecules have been explored; however, the interventions compromise the antimicrobial potency of the peptides. In a recent study, ADEP1 analogues showed poor antibacterial activity with a minimum inhibitory concentrations (MIC) of ≥63 μM against Gram-positive bacteria and ≥125 μM against Gram-negative bacterial strains [11]. These analogues are derivatives of highly potent ADEP1 (ADEP A54556A). The conjugation of such potential drug molecules to biocompatible nano-carriers, such as quantum dots (QDs), would increase the desired biological activity.

**Scheme 1:**
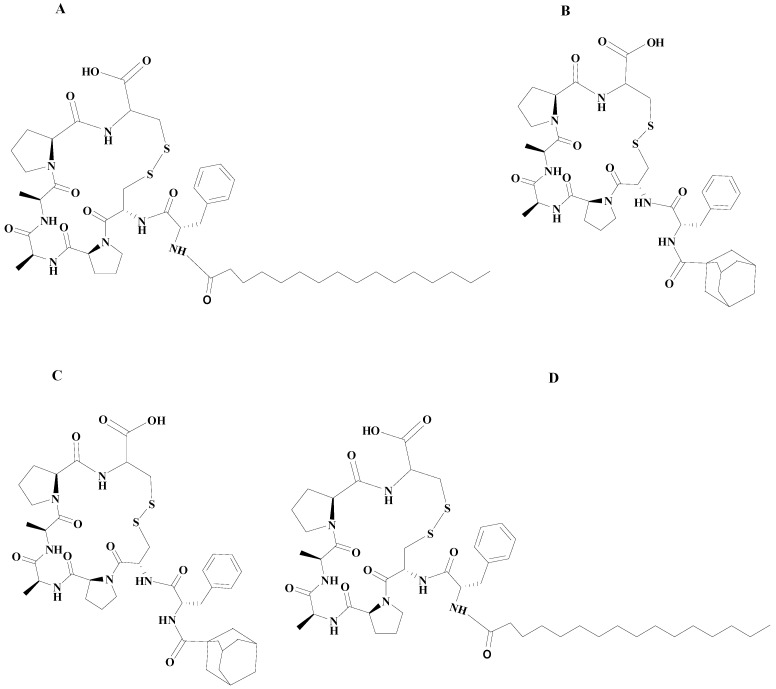
Disulphide bond cyclized ADEP1 analogues. (**A**)—SC005 (Palmitic acid-FCPAAPC; D-form amino acids); (**B**)—SC006 (Adamantane-FCPAAPC; D-form amino acids); (**C**)—SC007 (Adamantane-FCPAAPC; L-form amino acids); (**D**)—SC008 (Palmitic acid-FCPAAPC; L-form amino acids) [11].

QDs have gained traction in medicine as bio-imaging tools, drug delivery systems, and in-sensor applications [12]. QDs used as nano-carriers of drugs have been reported to improve bioavailability, biocompatibility, and the efficacy of drugs [13,14]. Conjugation of doxorubicin to ZnO QDs for the treatment of lung cancer is one example that highlights the effectiveness of using nanomaterials as drug delivery systems [15]. In this study, gelatin-capped Silver/Indium/Sulfide (AgInS_2_) QDs were utilized as nano-carriers for antimicrobial ADEP1 analogues, previously reported by Cobongela et al., [11]. AgInS_2_ QDs are known I-III-VI semiconductors that also exhibit a long-term photoluminescence decay lifetime [16]. Due to their remarkable photoluminescence properties and low cytotoxicity, they have found use in bio-imaging and cell targeting [17,18,19]. AgInS_2_ QDs were conjugated to ADEP1 analogues (Scheme 1) to improve their antibacterial efficacy.

## 2. Results and Discussion

### 2.1. Characterization of AgInS_2_ QDs

**Figure 1:**
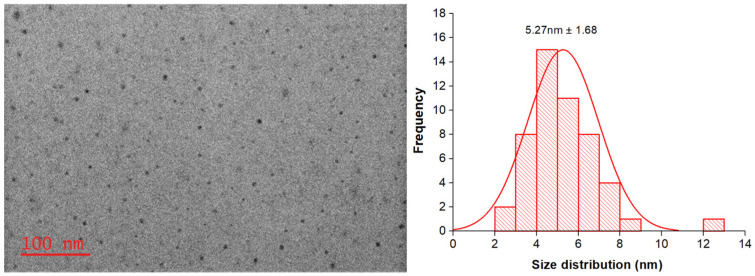
TEM image and size distribution of gelatin-capped AgInS_2_ QDs.

The HR-TEM in Figure 1 confirmed a successful synthesis of gelatin-capped AgInS_2_ QDs. The micrograph shows that the AgInS_2_ QDs are evenly dispersed and spherical in shape with a core size of 5.27 ± 1.68 nm. The elemental analysis of gelatin-capped AgInS_2_ QDs was performed using ED-XRF and confirmed the presence of Ag, In, and S; with their percentage and ratio shown in Table 1. The gelatin on the surface of the QDs accounted for the majority of the components of AgInS_2_ QDs.

### 2.2. Functionalization of AgInS_2_ QDs with ADEP1 Analogues

**Scheme 2:**
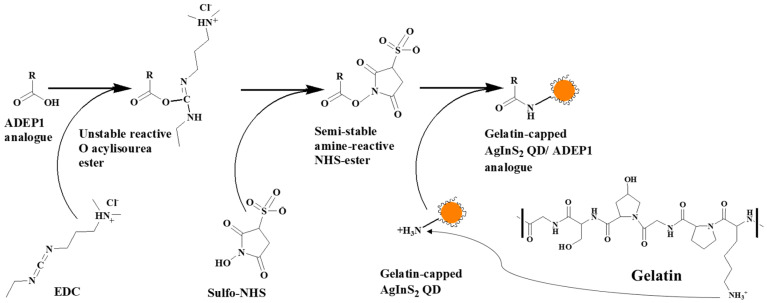
Conjugation of ADEP1 analogues to gelatin-capped AgInS_2_ QDs via EDC/sulfo-NHS coupling-chemistry. R = peptide sequence.

Gelatin-capped AgInS_2_ QDs were functionalized with ADEP1 analogues via EDC/sulfo-NHS coupling chemistry, as demonstrated in Scheme 2. The carboxylic groups of the ADEP1 analogues were activated in the absence of the gelatin-capped AgInS_2_ QDs. The gelatin contains an amine (NH_3_^+^) which is readily available to form an amide bond with the activated COO^−^ end of the ADEP1 analogues. Treatment of the ADEP1 analogues with EDC/sulfo-NHS in the presence of gelatin-capped AgInS_2_ QDs could potentially result in intramolecular coupling between these carboxylic groups and the amine groups on the gelatin. The conjugation utilized the gelatin lysine amino group on the surface of the AgInS_2_ QDs and the carboxylic group on the C-terminal of the ADEP1 analogues to form an amide bond [20].

**Figure 2:**
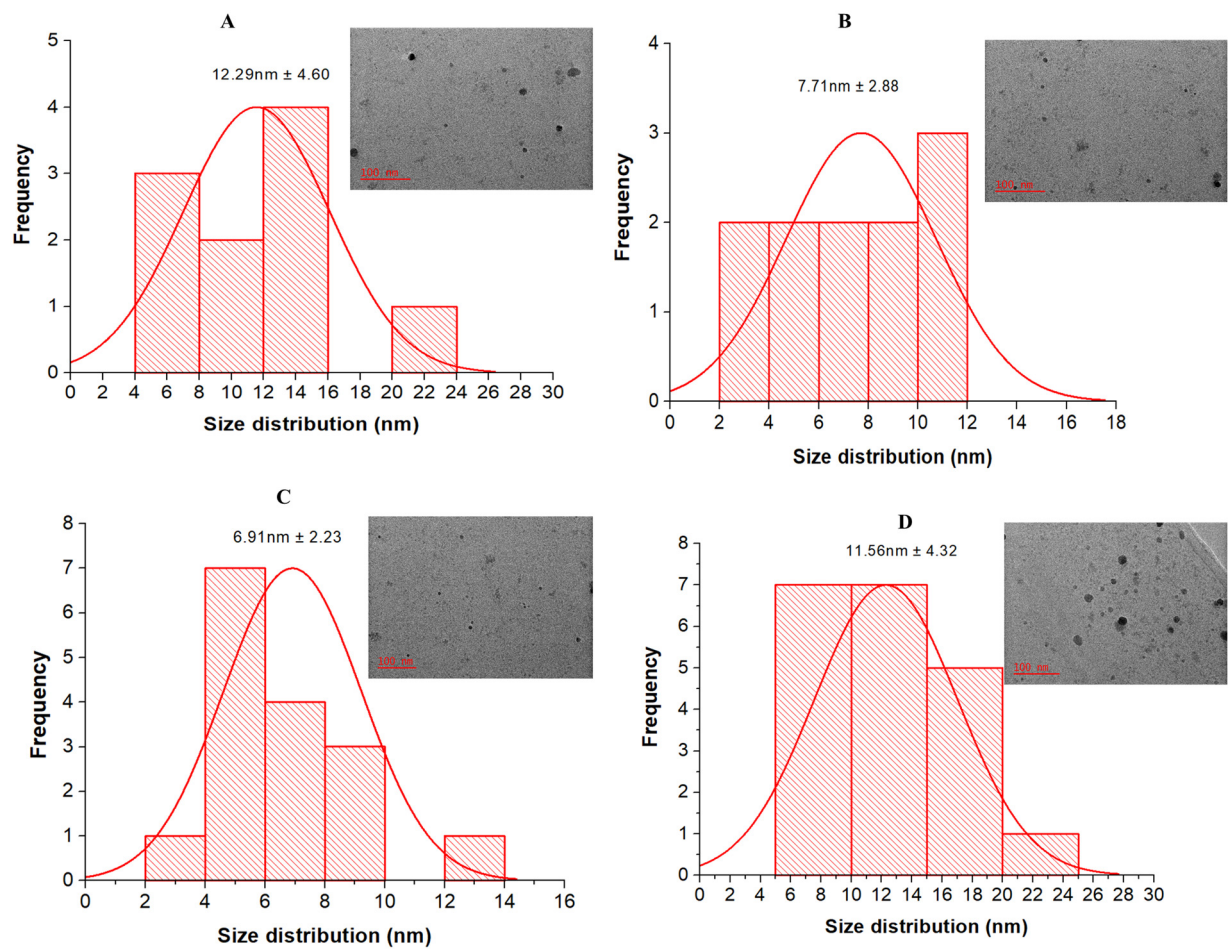
Core size distribution and TEM images of gelatin-capped AgInS_2_ QDs-ADEP1 analogue conjugates and their (**A**): AgInS_2_ QD-SC005; (**B**): AgInS_2_ QD-SC006; (**C**): AgInS_2_ QD-SC007; (**D**): AgInS_2_ QD-SC008.

**Figure 3:**
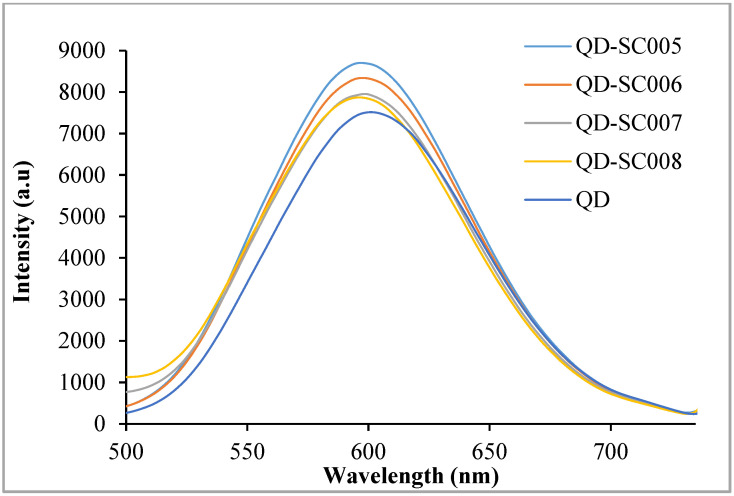
Photoluminescence properties of gelatin-capped AgInS_2_ QDs and AgInS_2_ QDs-ADEP1 analogue conjugates.

The conjugation of SC005-8 ADEP1 analogues increased the size of the AgInS_2_ QDs (Figure 2A–D). The size distribution of palmitic acid-containing (SC005 and SC008) conjugates changed to 11.6 and 12.3 nm, respectively, whereas the adamantane-containing (SC006 and SC007) conjugates had average size of 6.9 and 7.7 nm, respectively. The increase in the size distribution of the conjugates is an indication of changes on the surface of the QDs. Other studies have also noted an increase in nanomaterial and QD size upon conjugation with biomolecules, such as peptides [21,22,23]. Optical properties were measured using photoluminescence spectroscopy with emission wavelengths between 400 and 700 nm. Luminescent QDs, such as AgInS_2_ QDs, have long-lived excited states; therefore, the excitation wavelength was marginalized [24]. The gelatin-capped AgInS_2_ QDs had an emission peak at 603 nm while the AgInS_2_ QD-ADEP1 analogue conjugates shifted the emission to lower wavelengths. AgInS_2_ QDs conjugated to the pal-containing ADEP1 analogues (i.e., SC005 and SC008) absorbed at 595 nm while ada-containing ADEP1 analogues (i.e., SC006 and SC007) absorbed at 599 nm (Figure 3). Conjugation added a coating on the surface of the AgInS_2_ QDs, this in turn shielded the surface of the QDs. In addition, the shift in emission wavelength might be a result of increased size and the change in surface properties of the AgInS_2_ QDs post conjugation.

The AgInS_2_ QDs-ADEP1 analogue conjugates were further characterized using zetasizer and DLS to determine their surface charge and hydrodynamic size. Table 2 shows the zeta potential values of the ADEP1 analogues before and after conjugation. The AgInS_2_ QDs had a zeta potential value of −3.69 ± 0.9 mV. The ADEP1 analogues also had negative net charges ranging between −7.2 and −3.8 mV prior to conjugation. This confirmed the anionic state of the ADEP1 analogues, which is a result of the carboxylic anion (COO^−^) at physiological pH. Anionic peptides have a disadvantage in penetrating bacterial cells, as they are made up of negatively charged polysaccharides, while cationic peptides have been commended for their cell permeability capability [25]. ADEP1 analogues lack the cationic advantage, which may lead to poor cell permeability and bioavailability. Therefore, to mitigate the challenges of poor pharmacokinetic properties associated with ADEPs, the analogues were conjugated to AgInS_2_ QDs. The DLS results showed an increased diameter of AgInS_2_ QDs (36.7 ± 2.8) nm compared to the particle diameter measured by TEM. The conjugation also led to an increased hydrodynamic diameter of the AgInS_2_ QDs (Table 2), with palmitic acid-containing ADEP1 analogues being slightly bigger than the adamantane-containing ADEP1 analogues. These results showed that the conjugation of the ADEP1 analogues to AgInS_2_ QDs resulted in a positive net charge, which could enhance the cell permeability properties of the peptides.

**Figure 4:**
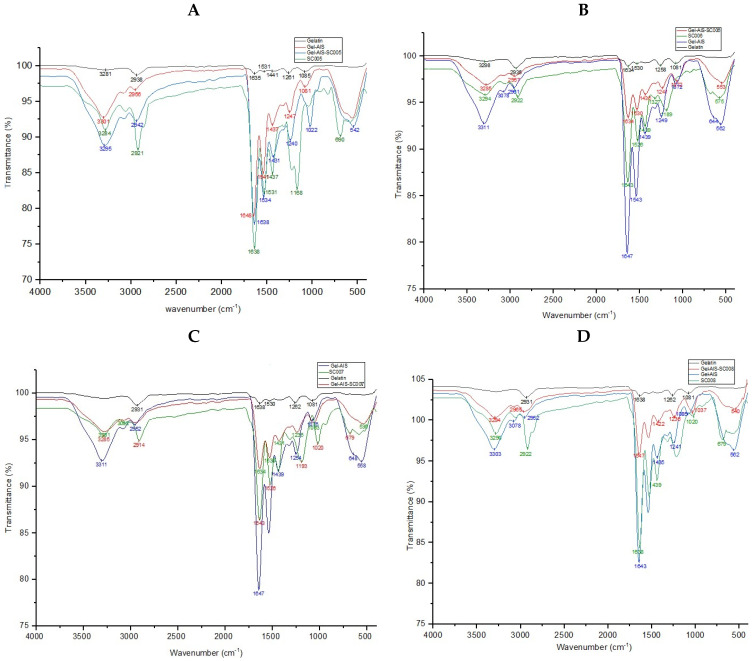
FTIR analysis of functional groups involved in gelatin capping of AgInS_2_ QDs, and AgInS_2_ QDs conjuction with ADEP1 analogues. (**A**) SC005, (**B**) SC006, (**C**) SC007 and (**D**) SC008.

The FTIR spectra revealed the functional groups present on gelatin, the surface of the AgInS_2_ QDs, ADEP1 analogues, and AgInS_2_ QDs-ADEP1 analogue conjugates (Figure 4). There were similarities and shifts in the peaks of these molecules that suggested that gelatin was part of the AgInS_2_ QDs, and that the ADEP1 analogues were successfully incorporated in the AgInS_2_ QDs.

As shown in Table 3, gelatin showed broad associated N-H_str_ absorption signals for secondary (2°) amine between 3700 and 3000, peaking at 3300 cm^−1^, small sharp peaks at 2860 and 2920 cm^−1^ for symmetry and asymmetry saturated hydrocarbon (C-H_str_), small multiplet signals between 1700 and 1000 cm^−1^ for C=O_str_ (amide band I and II, 1625, 1525, respectively), 1450 and 1275 cm^−1^ for C-N_str_, 1050 cm^−1^ for C-O_str_ and the absence of peaks between 1000 and 500 cm^−1^. After the interaction of ADEP1 analogues with AgInS_2_, a new peak appeared at 575 cm^−1^ with a shoulder at 675 cm^−1^, corresponding to N-H_bend_ out-of-plane, implying complexation with the metal ions on the surface of the QDs. The increase in the intensities of the amino (N-H) and carbonyl (C=O) groups and a shift to a higher frequency (wavenumber) in AgInS_2_ QDs-ADEP1 analogue conjugates further confirmed the complexation of the QDs with the ADEP1 analogues. Generally, after conjugation of the ADEP1 analogues to the QDs, peak intensities of all functional groups associated with AgInS_2_ QDs were reduced. The initial C-H_str_, C-O_str_, and N-H_bend_ peaks of the ADEP analogues were also replaced with those of the AgInS_2_ QDs. All of this information confirmed the coupling of the AgInS_2_ QDs with the ADEP1 analogues to form the hybrid material.

### 2.3. Antibacterial Activity of AgInS_2_ QDs-ADEP1 Analogue Conjugates

Following the successful synthesis of the gelatin-capped AgInS_2_ QDs and their subsequent conjugation to the ADEP1 analogues, the antibacterial activity of the conjugates was tested against three Gram-positive (*B. subtilis*, *S. aureus* and Methicillin-resistant *S. aureus*) and two Gram-negative (*E. coli* and *P. aeruginosa*) bacterial strains. The MICs and MBCs of the AgInS_2_ QDs-ADEP1 analogue conjugates were evaluated. On their own, the AgInS_2_ QDs did not show any antibacterial activity against the selected bacterial strains at the tested concentrations. The ADEP1 analogues, as reported in [11], had MIC and MBC values that were between 63 and 500 µM and 125 and 750 µM, respectively. However, upon conjugation to the ADEP1 analogues, the antibacterial activity was significantly improved. The MIC and MBC values of the AgInS_2_ QDs-ADEP1 analogue conjugates on the tested strains ranged from 1.6 to 25 µM and 6.3 to 100 µM, respectively (Table 4).

The most susceptible bacterium to the AgInS_2_ QD-ADEP1 analogue conjugates was *E. coli* with an MIC of 1.6 µM, followed by *B. subtilis* and *S. aureus* with MIC values of 6.3 µM, MRSA at 12.5 µM and lastly, *P. aeruginosa* at 25 µM. These results showed that the AgInS_2_ QD-ADEP1 analogue conjugates were effective against both Gram-positive and Gram-negative bacteria. A study conducted by Cobongela et al. [11] reported that the Gram-negative bacterial strains (*E. coli* and *P. aeruginosa*) showed resistance against the ADEP1 analogues with MIC and MBC values of 500 and 750 µM, respectively. These observations were in line with previous studies, which reported a higher ADEP1 activity against Gram-positive bacteria compared to Gram-negative bacteria [26]. These new findings showed that conjugating the ADEP1 analogues to the AgInS_2_ QDs helped overcome the resistance that was exhibited by *E. coli* against these peptides. Previous studies have discovered that peptides conjugated to nanomaterials show increased activity due to a re-binding mechanism to the target, which then increases the retention time of the peptide within the target [27]. The re-binding or reassociation mechanisms of nano-conjugates is enhanced by the slow dissociation rate of conjugates compared to free drug molecules. In addition, the increased density of the conjugate compared to either the nanomaterial or peptide alone exhibits increased binding affinity due to steric hindrance introduced upon binding to the target [28,29].

### 2.4. Cytotoxicity Screening of AgInS_2_ QDs-ADEP1 Analogue Conjugates

**Figure 5:**
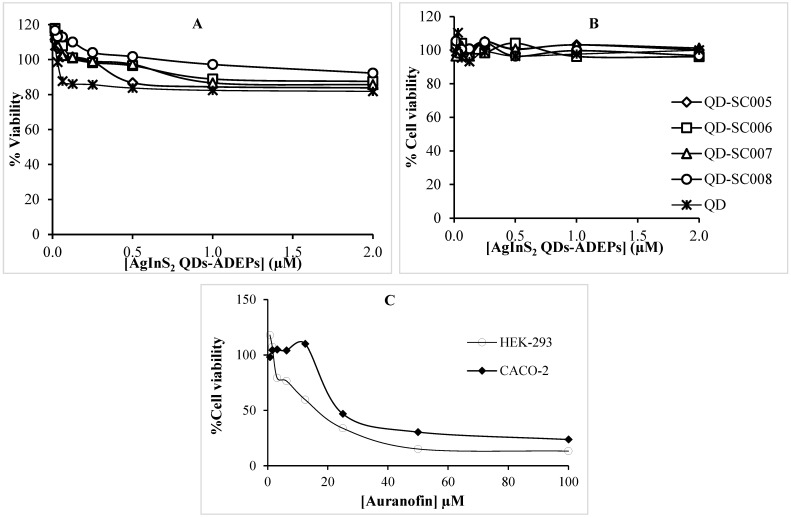
Effect of AgInS_2_ QD-ADEP1 analogue conjugates (**A**) on HEK-293 and (**B**) Caco-2 cell viability. Cells treated with auranofin (**C**) were used as a positive control.

The cytotoxicity of the AgInS_2_ QDs-ADEP1 analogue conjugates was evaluated on HEK-293 cells using the MTS assay. The HEK-293 cells originate from the kidney, which is one of the organs in the excretory or renal system and is responsible for the removal of unwanted or toxic materials from the body [30]. Evaluating cytotoxicity is of utmost importance, especially if the materials are to be used in biological applications. For this assay, the HEK-293 cells were exposed to the AgInS_2_ QDs-ADEP1 analogue conjugates (loaded with ~1.2–150 µM of ADEP1 analogues) for 72 h. The results presented in Figure 5A show cell viability greater than 80% for all of the tested concentrations of the AgInS_2_ QDs and AgInS_2_ QDs-ADEP1 analogue conjugates. In a study by Oluwafemu et al., a similar trend in cell viability was observed when the similar QDs variant, AgInS_2_/ZnS core/shell, were tested against baby hamster kidney cells [18]. The AgInS_2_ QDs used in the current study are regarded environmentally friendly as they are capped with gelatin, an animal product that is also used in food.

## 3. Materials and Methods

### 3.1. Materials

ADEP1 analogues (SC005-8) were synthesized in-house, as described by Cobongela et al., [11]. 1-Ethyl-3-(3-dimethylaminopropyl) carbodiimide (EDC), N-hydroxysuccinimide (sulfo-NHS), dimethyl sulfoxide (DMSO), thioglycolic acid (TGA), silver nitrate (AgNO_3_), sodium sulfide (Na_2_S·*x*H_2_O), gelatin, Dulbecco’s Modified Eagle Medium (DMEM), phosphate, trypsin, penicillin-streptomycin, fetal bovine serum (FBS), 3-(4,5-dimethylthiazol-2-yl)-5-(3-carboxymethoxyphenyl)-2-(4-sulfophenyl)-2H-tetrazolium (MTS dye), and trypan blue stain dye were all purchased from Sigma (St Louis, MO, USA). *Bacillus subtilis* American Type Culture Collection (ATCC) 11774, *Staphylococcus aureus* ATCC 25,923 and Methicillin-resistant *Staphylococcus aureus* (MRSA) ATCC 43300, and two Gram-negative (*Escherichia coli* ATCC 33,876 and *Pseudomonas aeruginosa* ATCC 15,442 were purchased from ATCC (Manassas, VA, USA). Human embryonic kidney 293 (HEK-293) cells were purchased from Cellonex (Randburg, Gauteng Province, South Africa).

### 3.2. Synthesis of AgInS_2_ QDs

The AgInS_2_ QDs were synthesized using a procedure reported by May et al., 2019 [31], with some modifications. In a typical synthesis, 0.25 mmol (0.0753 g) of In (NO_3_)_3_, 0.438 mmol TGA, 0.0625 mmol of silver nitrate, 0.2941 g of gelatin, and 0.406 mmol of Na_2_S.9H_2_0 were added to 100 mL of deionized water with stirring (Ag:In:S:gelatin:TGA mole ratio of 1:4:6.5:16:7, respectively). The pH was adjusted to 6. The mixture was refluxed at 95 °C for 1 h to produce the gelatin-capped AgInS_2_ QDs. The QDs were precipitated with ethanol and air dried.

### 3.3. Conjugation of AgInS_2_ QDs to ADEP1 Analogues

The ADEP1 analogues were synthesized using the Fmoc solid peptide synthesis strategy, as previously described [11]. ADEP1 analogues (600 µM) dissolved in 5 mL of dimethyl formamide were treated with EDC (0.75 µM) and sulfo-NHS (0.3 µM), and the reaction was left shaking for 3 h at room temperature. Concurrently, 8 mg/mL of AgInS_2_ QDs were dissolved in 10 mL of boiling water. Upon cooling down, the AgInS_2_ QDs were added to the ADEP1 analogues solution, and the reaction was left shaking for 3 h at room temperature. The AgInS_2_-ADEP1 analogue conjugates were precipitated with ethanol and lyophilized (LyoQuest, Telstar, Terrassa, Barcelona). The powder was re-suspended in phosphate buffered saline (PBS; pH 7.4) to a final concentration of 4 mg/mL AgInS_2_-ADEP1 analogue conjugates with 200 µM ADEP1 analogues.

### 3.4. Characterization of QDs, AgInS_2_-ADEP1 Analogue Conjugates, and ADEP1 Analogues

A Spectro Xepos-05 energy-dispersive X-ray fluorescence (ED-XRF, Rigaku, TX, USA) instrument was used to determine the Ag, In, and S concentrations in the gelatin-capped AgInS_2_ QD powder. The core sizes of AgInS_2_ QDs and AgInS_2_-ADEP1 analogue conjugates were analyzed by the high-resolution transmission electron microscope (HR-TEM, JEOL, Tokyo, Japan). Fourier transform infrared spectroscopy (FTIR) spectra were recorded using a Perkin Elmer Spectrum Two UATR-FTIR spectrometer (PerkinElmer, Buckinghamshire, UK). The Hitachi F-2700FL spectrofluorophotometer (Hitachi, Tokyo, Japan) was used for the fluorescence measurements. Malvern system 4700 zetasizer (Malvern, Great Malvern, UK) together with a dynamic light scattering (DLS) technique were used to determine the zeta potential and particle hydrodynamic size of the AgInS_2_ QDs and AgInS_2_-ADEP1 analogue conjugates using. 

### 3.5. Antibacterial Activity of AgInS_2_-ADEP1 Analogue Conjugates

The MIC and MBC of the AgInS_2_-ADEP1 analogue conjugates against *B. subtilis*, *S. aureus*, MRSA, *P. aeruginosa* and *E. coli* was determined using the broth microdilution susceptibility assay, following a previously described protocol [11]. Briefly, overnight-grown bacterial cultures were adjusted to approximately 1.5 × 10^8^ CFU/mL in a sterile Luria–Bertani broth. The concentration of ADEP1 on AgInS_2_-ADEP1 analogue conjugates was calculated based on the ADEP1 analogues calibration curve. AgInS_2_-ADEP1 analogue conjugates and ADEP1 analogues were diluted via 2-fold serial dilution with broth containing a final concentration range of 0.8–100 µM and 15.6–2000 µM, respectively. Gentamicin (concentration range between 0.007 and 6.7 mM) was used as a positive control for *B. subtilis*, *S. aureus*, MRSA, and *P. aeruginosa*, while ampicillin (concentration range between 0.18 and 6.7 mM) was used as a positive control for *E. coli*, and 5% DMSO was used as a negative control. The 96-well plates were then incubated overnight at 37 °C. The lowest concentration of the AgInS_2_-ADEP1 analogue conjugates and controls that resulted in clear wells with no turbidity (i.e., no visible bacterial growth) was recorded as the MIC. The bacterial culture from the MIC wells at concentrations of AgInS_2_-ADEP1 conjugates above the MIC were inoculated into Luria–Bertani agar plates and incubated overnight at 37 °C. The MBC was determined as the lowest concentration resulting in complete bacterial-growth inhibition.

### 3.6. Cytotoxicity Activity of AgInS_2_-ADEP1 Analogue Conjugates

The cytotoxicity of the AgInS_2_-ADEP1 analogue conjugates was assessed on HEK-293 cells (purchased from Cellonex (Randburg, Gauteng Province, South Africa)) using the MTS cell proliferation assay, as previously reported [11]. The cells were cultured in Dulbecco’s Modified Eagle Medium (DMEM) consisting of 10% fetal bovine serum (FBS), 2% DMSO, and 1% penicillin–streptomycin. The cells were seeded at 1 × 10^5^ cells/mL into a 96-well plate and incubated at 37 °C overnight in a humidified incubator at 5% CO_2_. The cells were then treated with AgInS_2_-ADEP1 analogue conjugates to attain a final concentration range of 1.2–150 µM. Auranofin was used as a positive control at a concentration range of 0.78–100 µM and DMSO (2%) was used as a negative control. After 72 h of treatment, the metabolic activity of cells was determined by adding 10% of MTS dye. The percentage cell viability was calculated using the equation below:

(1) $$\%\;cell\;viability=\left(\frac{OD\;of\;test\;sample}{OD\;of\;control}\right)\times 100$$

## 4. Conclusions

In this study, gelatin-capped AgInS_2_ QDs were synthesized and conjugated to novel ADEP1 analogues, with the aim of improving the antibacterial efficacy of these peptides. The conjugation of the QDs to the peptides was confirmed using multiple techniques, namely, zeta potential, PL, ED-XRF, and HR-TEM. Compared to the AgInS_2_ QDs, which did not show any appreciable antibacterial activity, and the ADEP1 analogues, which presented moderate activity, the AgInS_2_ QDs-ADEP1 analogue conjugates exhibited excellent antibacterial activity. The MIC values obtained for the AgInS_2_ QDs-ADEP1 analogue conjugates were between 3- and 200-folds lower than those obtained for the ADEP1 analogues, while the MBC values were between 5- and 13-folds lower. The MIC and MBC values showed that the AgInS_2_ QDs-ADEP1 analogue conjugates were mostly potent against *E. coli*, followed by *B. subtilis* and *S. aureus*. The AgInS_2_ QDs-ADEP1 analogue conjugates were non-toxic to HEk-293 cells at concentrations higher than the MIC and MBC and can be considered to be biocompatible. These findings suggest that the AgInS_2_ QDs-ADEP1 analogue conjugates could potentially be used as antibacterial agents without eliciting any significant toxic effects against mammalian cells. The photoluminescence and fluorescent properties of AgInS_2_ QDs will be exploited in future investigations to monitor ADEP1 analogues targeting, delivery and response in real time, and further determine the antibacterial mode of action of these ADEP1 analogues.

## Figures and Tables

**Table 1:** Elemental analysis of gelatin-capped AgInS_2_ QDs.

Elements	Ag	In	S
%	0.8	1.6	14.4
Ratio	1	2	18

**Table 2:** Zeta potential values and hydrodynamic size of the ADEP1 analogues before and after conjugation to AgInS_2_ QDs.

ADEP1 Analogue	Zeta Potential (mV) before Conjugation	Zeta Potential (mV) after Conjugation	Hydrodynamic Diameter (nm)
AgInS_2_ QDs	−3.7 ± 0.9	-	36.7 ± 2.8
SC005	−7.2 ± 1.6	1.46 ± 0.02	49.6 ± 1.6
SC006	−3.8 ± 1.1	1.38 ± 0.06	44.2 ± 1.4
SC007	−3.9 ± 0.8	1.00 ± 0.15	44.4 ± 0.7
SC008	−6.9 ± 1.5	1.39 ± 0.09	50.3 ± 1.6

**Table 3:** FTIR data for gelatin, AgInS_2_ QDs and AgInS_2_ QDs-ADEP1 analogue conjugates.

Functional Groups	Gelatin	AgInS_2_-QDs	ADEP1 Analogues	AgInS_2_ QDs-ADEP1 Analogues
N-Hstr	3300	3300	3300	3300
C-Hstr	2920–2860	2960–2875	2920–2860	2960–2875
C=Ostr (Amide band I, Amide band II)	1625, 1525	1637, 1537	1625, 1525	1637, 1537
C-Nstr	1450, 1390	1450, 1400	1450	1450, 1400
C-Nstr	1275, 1390	1237, 1337	1237, 1337	1237, 1337
C-Ostr	1050	1090	1190	1090
N-Hbend (out-of-plane)	x	575, 675	700	575, 675

**Table 4:** MIC and MBC values of the AgInS_2_ QD-ADEP1 analogue conjugates and ADEP1 analogues.

Treatments	*B. subtilis*		*S. aureus*		MRSA		*P. aeruginosa*		*E. coli*	
	MIC µM	MBC µM	MIC µM	MBC µM	MIC µM	MBC µM	MIC µM	MBC µM	MIC µM	MBC µM
QD-SC005 SC005	6.3 125	25.0 125	6.3 63	25.0 125	12.5 125	50.0 250	25.0 125	100.0 250	1.6 500	6.3 750
QD-SC006 SC006	6.3 250	25.0 250	6.3 63	25.0 125	12.5 250	50.0 250	25.0 125	100.0 250	1.6 500	6.3 750
QD-SC007 SC007	6.3 125	25.0 125	6.3 63	25.0 125	12.5 250	50.0 500	25.0 125	100.0 250	1.6 500	6.3 750
QD-SC008 SC008	6.3 125	25.0 125	6.3 63	25.0 125	12.5 250	50.0 500	25.0 125	100.0 250	1.6 500	6.3 750
Gentamicin	26.1	52.3	0.5	1.0	26.2	52.4	419	838	NT	NT
Ampicillin	NT	NT	NT	NT	NT	NT	NT	NT	2862	5724

## Data Availability

The data in this study are presented as tables and figures.
